# Endocarditis Masquerading as Malignancy: Native Valve HACEK Infection

**DOI:** 10.1016/j.jaccas.2026.108554

**Published:** 2026-06-24

**Authors:** Vlad S. Shusterman, Gidon Salamatbad, Andrew Cyr, Yisrael Wallach, Alexander Carlin, Christine Yen, Eileen Kim, Andrew Galmer

**Affiliations:** a Department of Internal Medicine, Northwell North Shore University Hospital, Manhasset, New York, USA; b Department of Cardiology, Northwell North Shore University Hospital, Manhasset, New York, USA

**Keywords:** atrial fibrillation, endocarditis, echocardiography

## Abstract

**Background:**

HACEK (Haemophilus, Aggregatibacter, Cardiobacterium, Eikenella, Kingella) endocarditis is a rare cause of subacute infective endocarditis typically occurring in patients with pre-existing valvular pathology or structural heart disease.

**Case Summary:**

A 65-year-old male presented with months of night sweats, fevers, anemia, and weight loss concerning for malignancy. Transthoracic echocardiography was negative for vegetations. Blood cultures grew *Aggregatibacter actinomycetemcomitans*, and transesophageal echocardiography confirmed vegetations on the mitral and aortic valves. The patient was treated successfully with a 6-week course of intravenous ceftriaxone.

**Discussion:**

This case highlights the diagnostic challenge of indolent HACEK endocarditis in the absence of traditional risk factors, presenting as malignancy, underscoring the importance of transesophageal echocardiography, even when transthoracic imaging is unrevealing.

**Take-Home Message:**

In patients with persistent constitutional symptoms with negative malignancy workup, HACEK endocarditis should remain in the differential, and transesophageal echocardiography is essential for diagnosis.

## Visual Summary

**Figure:**
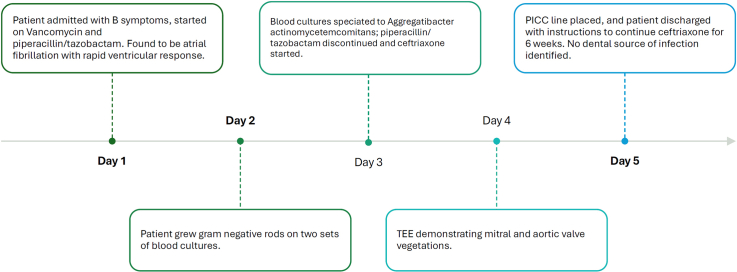

**Visual Summary:**
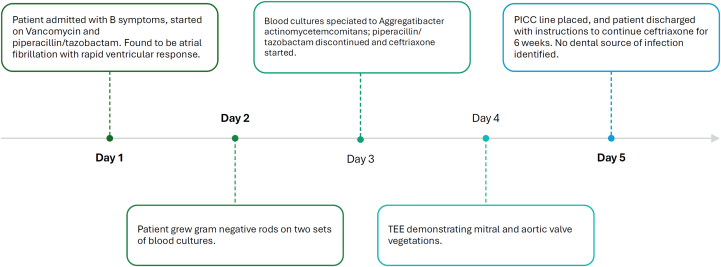
Timeline of Clinical Presentation, Diagnosis, and Management PICC = peripherally inserted central catheter; TEE = transesophageal echocardiogram.

## History of Presentation/Past Medical History

A 65-year-old male, with past medical history of hypertension, hyperlipidemia, diabetes mellitus, and thyroid mass, presented after a near-syncopal event at night when getting out of bed to use the restroom accompanied by fevers and chills. Four months prior to admission, he complained of night sweats/fevers and 20-pound weight loss, with an extensive malignancy workup as outpatient, which revealed a normocytic anemia, negative colonoscopy, negative prostate-specific antigen, imaging negative for lymphoma, and normal bone marrow biopsy. A biopsy of the thyroid mass revealed benign follicular nodular tissue.

In the emergency room, temperature was 101.5 F, oxygen saturation >95% on room air, and electrocardiogram revealed atrial fibrillation with rapid ventricular response (Figure 1) at a rate of 163 with a blood pressure of 80/50. The patient received fluid resuscitation and was started on phenylephrine drip. He met sepsis criteria and was started on vancomycin and piperacillin/tazobactam, as well as amiodarone and heparin drip. Infectious workup, including respiratory viral panel, blood cultures, and urine cultures, was drawn. In addition, transthoracic echocardiogram (TTE) showed normal left ventricular ejection fraction of 50% to 55%, with no evidence of vegetations, and demonstrated mild mitral regurgitation (Video 1, Video 2, Video 3, Video 4, Video 5). The patient was successfully weaned from phenylephrine drip and returned to normal sinus rhythm (Figure 2), after which he was admitted to medicine for further workup. Neither electrocardiogram demonstrated conductivity abnormalities suggestive of perivalvular abscess.

**Figure 1:**
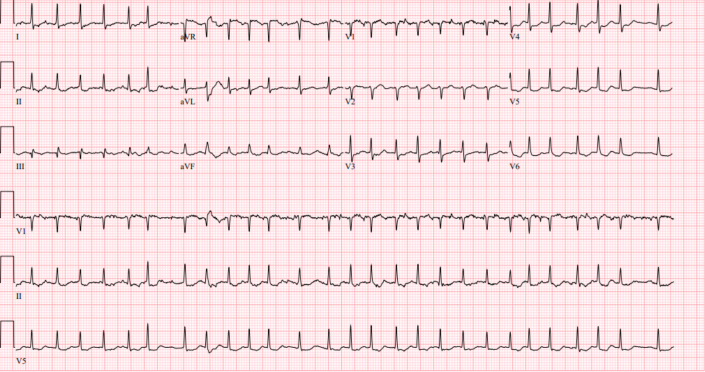
Initial Electrocardiogram Demonstrating Atrial Fibrillation With Rapid Ventricular Response (Heart Rate of 163 beats/min), Prior to Fluid Resuscitation

**Figure 2:**
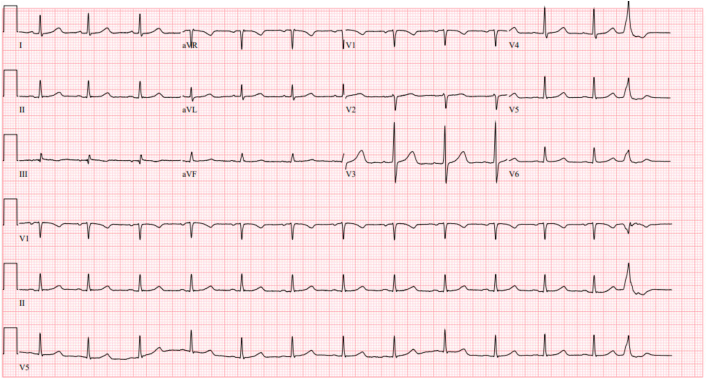
Electrocardiogram Demonstrating Restoration of Normal Sinus Rhythm With Premature Ventricular Complexes, Following Amiodarone, Antibiotic Initiation, and Fluid Resuscitation (Heart Rate 80 beats/min)

The patient has several family members with Hashimoto’s disease and no other pertinent family history. He had no surgical history and no known drug allergies. Social history was positive for social drinking and a 15 pack-year smoking history prior to quitting 20 years ago.

## Differential Diagnosis

At this point, differential diagnosis included malignancy (lymphoma, gastric cancer, etc), autoimmune/autoinflammatory conditions, or smoldering infection such as tuberculosis.

On physical examination, repeat vital signs were stable. The patient was euvolemic appearing, and no jugular venous distension or hepatojugular reflex was noted. Lungs were clear to auscultation bilaterally. On cardiac auscultation, normal rate and rhythm with no murmurs, rubs, or gallops were observed. No Janeway or Osler nodes were seen. No swelling of the lower extremities was observed. Neurological physical examination was within normal limits.

After the initiation of antibiotic therapy, the patient reported feeling well, no longer endorsing any fevers, chills, or body aches. A nasal swab for Methicillin-resistant *Staphylococcus aureus* culture returned negative, and vancomycin was discontinued, leaving only piperacillin/tazobactam for empiric coverage.

Given the subacute chronology of patient’s symptoms, as well as strong family history of autoimmune disease, rheumatological causes of his symptoms were considered; however, autoimmune workup was largely noncontributory. To complete infectious workup, imaging of the abdomen/pelvis was ordered to rule out occult infection, which came back unremarkable.

## Investigations

After 48 hours, blood cultures grew gram-negative rods. A tagged white blood scan was ordered, which did not reveal any source of infection (Figure 3), highlighting the diagnostic challenge of his constitutional symptoms. The next day, the cultures speciated to *Aggregatibacter actinomycetemcomitans*. The patient was switched to ceftriaxone and had transesophageal echocardiogram (TEE) ordered, which revealed mobile vegetations in the anterior and posterior mitral valve leaflets, as well as a small echodensity suggestive of aortic valve vegetation (Video 6, Video 7, Video 8, Video 9, Video 10, Figures 4 and 5).

**Figure 3:**
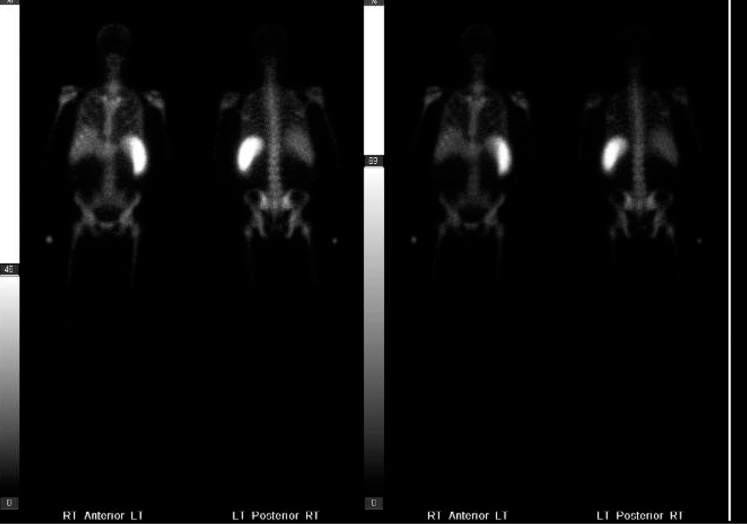
Tagged White Blood Cell Scan Demonstrating Physiological Spleen, Liver, and Bone Marrow Enhancement, With No Evidence of Infection

**Figure 4:**
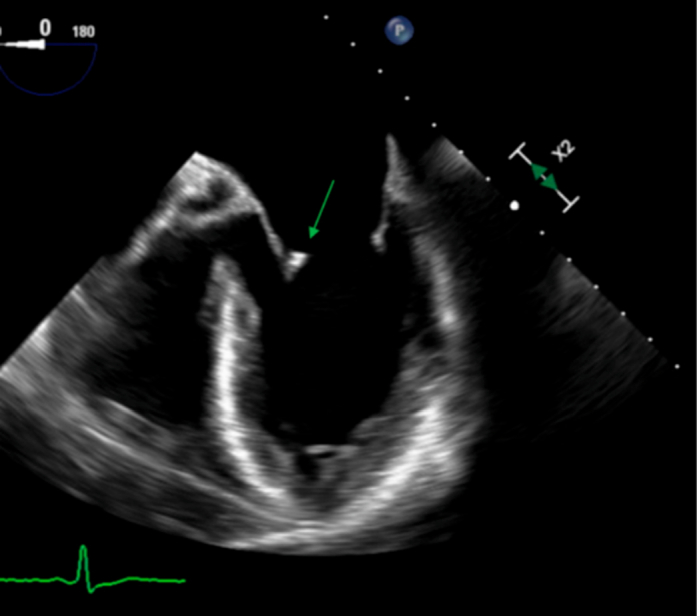
Transesophageal Echocardiography Demonstrating a Structure Consistent With a Vegetation on the Anterior Mitral Valve Leaflet, as Indicated by the Green Arrow

**Figure 5:**
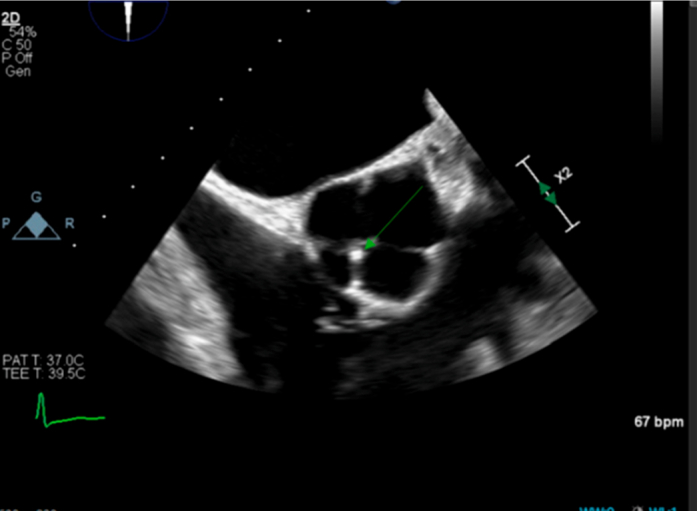
Transesophageal Echocardiography Demonstrating a Hyperechoic Density Over the Right Coronary Cusp, Consistent With Vegetation, as Indicated by the Green Arrow

## Management

A peripherally inserted central catheter line was placed for prolonged intravenous antibiotic therapy, for a total of 6 weeks of therapy with ceftriaxone. Cardiothoracic surgery was consulted and determined that no surgical intervention was required. Prior to discharge, the patient had a dental evaluation, which showed no evidence of odontogenic infection, and per the evaluation, dental infections were an unlikely cause of his infective endocarditis (IE). However, the patient did endorse losing a tooth 2 to 3 weeks prior to hospital admission, notably after the majority of his symptoms started.

## Outcome and Follow-Up

The patient remained in sinus rhythm during the entirety of his admission and was discharged to follow-up with infectious disease, cardiology for repeat TEE, and repeat dental evaluation. However, unfortunately, the patient was lost to follow-up. A visual timeline summarizing the clinical course can be seen in Figure 6.

## Discussion

IE is an infection of the endocardial surface of the heart, with numerous systemic consequences that are often life-threatening. According to the American College of Cardiology/American Heart Association guidelines from 2020, IE is diagnosed using the modified Duke’s criteria, which includes either 2 major criteria, 1 major and 3 minor criteria, or 5 minor criteria.^1^ Major criteria are either positive blood cultures of organisms known to cause endocarditis or imaging concerning for intracardiac vegetations or oscillating cardiac masses, whereas minor criteria include pre-existing cardiac conditions, fever, vascular phenomena, immunologic phenomena, or other microbiological evidence.^1^ Our patient met 2 major criteria, and thus met the guideline definition of IE. However, diagnosis is not always straightforward despite recent advancements such as blood cultures and echocardiography. Although less common than gram-positive species, IE can be caused by HACEK (Haemophilus, Aggregatibacter, Cardiobacterium, Eikenella, Kingella) organisms, which usually are part of the normal human oral/upper respiratory flora, and can cause a more insidious onset of endocarditis, usually affecting patients with either prosthetic valves, diseased native valves, or pre-existing heart disease.^2^^,^^3^ In North America, these organisms make up only 0.3% of all IE causes.^2^ However, despite the fact that most patients will achieve clinical resolution with treatment, HACEK endocarditis is more likely to result in cardioembolic, vascular, and immunologic complications, therefore should be identified and treated promptly.^4^

*A. actinomycetemcomitans* is a common bacterium that is found inside the oral microbiome in humans and is a common cause of periodontal disease.^5^^,^^6^ However, as an invasive pathogen, it is known to cause endocarditis, septic arthritis, meningitis, osteomyelitis, and empyema.^5^ Of all the HACEK organisms, it is the most common bacteria to cause IE.^7^ *A. actinomycetemicomitans* leukotoxin is a potent defense against white blood cells,^5^^,^^6^^,^^8^ and these proteins might play a role in lysing red blood cells as well, leading to hemolytic destruction,^8^ potentially explaining the patient’s anemia. The usual treatment for *A. actinomycetemcomitans* IE involves 4 to 6 weeks of a third-generation cephalosporin.^4^^,^^5^ Given increasing antibiotic resistance, future therapies involve research into leukotoxin A inhibitors, which would disinhibit the natural defense of the bacteria against leukocytes.^5^

Guidelines currently recommend TTE to evaluate for vegetations; however, if the TTE is not diagnostic, and suspicion of IE is high enough, TEE is a reasonable next step as a class 1 indication. TEE is especially helpful in patients with native valves, as the sensitivity is considered 90% to 100% in native valve endocarditis cases for TEE.^1^

The patient presented in this case report is unusual for several reasons. First, the patient presented in this report initially appeared to present with constitutional symptoms concerning for malignancy; however, after no malignancy was identified following careful investigation, it was determined that the patient had HACEK endocarditis. While TTE was negative for vegetations, given that blood cultures grew *A. actinomycetemcomitans*, TEE was indicated based on the current guidelines, and the TEE was able to visualize the vegetations on 2 valves which ultimately led to a successful diagnosis. This case reinforces the importance of TEE for cases that mimic malignancy, with no positive malignancy workup.

Perhaps the most unique aspect of the presented case is that it is uncommon for HACEK endocarditis to present with healthy native valves or without pre-existing cardiac conditions. For instance, Yukuhira et al describe a case of a 62-year-old male presenting with pyogenic spondylitis, who likely also had IE, given blood cultures growing the bacteria and spinal infection; however, the patient had periodontal infection, confirmed by a dentist, and a history of aortic valve replacement surgery.^9^ A case reported published by Borcan et al^10^ highlights a patient with native valves; however, that patient had a history of patent ductus arteriosus and poor dental hygiene, as well as chronic alcohol use, leading to altered mental state, another risk factor for bacterial translocation.^10^ That particular patient also had pulmonary valve vegetations and subsequent septic pulmonary emboli, whereas our patient had a much more favorable course despite having 2 valves affected. Although our patient was lost to follow-up, he ended up recovering well during his hospital admission.

Future research into native valve HACEK endocarditis would benefit from identifying patients at risk, and can possibly involve a multidisciplinary approach between cardiology, infectious disease, and dentists to identify patients early before complications like stroke arise, and promoting alternative therapies like leukotoxin A inhibitors to avoid antimicrobial resistance and drug-resistant microbes.

## Conclusions

This case highlights indolent IE due to *A. actinomycetemcomitans* involving a native valve that initially presented with constitutional symptoms concerning for malignancy such as lymphoma. It underscores the importance of considering IE when malignancy workup is unrevealing and reinforces the pivotal role that transesophageal echocardiography plays when transthoracic echocardiography is nondiagnostic for valvular vegetation despite high clinical suspicion.

## Funding Support and Author Disclosures

The authors have reported that they have no relationships relevant to the contents of this paper to disclose.

**Take-Home Message**

• In patients with persistent constitutional symptoms with negative malignancy workup, HACEK endocarditis should remain in the differential, and transesophageal echocardiography is essential for diagnosis.

## Appendix

Video 1 Transthoracic Echocardiography (Parasternal Long Axis) Demonstrating Normal Ejection Fraction Without Visible Valvular Vegetations Mitral leaflet thickening seen, along with calcifications along aortic valve.

Video 2 Transthoracic Echocardiography (Parasternal Long Axis) with Color Doppler Demonstrating Evidence of Mild Mitral Regurgitation

Video 3 Transthoracic Echocardiography (Apical 4-Chamber View) Demonstrating Normal Ejection Fraction No valvular vegetations seen despite clinical suspicion for infective endocarditis.

Video 4 Transthoracic Echocardiography (Apical 4-Chamber View With Mitral Valve Zoom) With No Valvular Vegetations Demonstrated

Video 5 Transthoracic Echocardiography (Apical 4-Chamber View) With Color Doppler Demonstrating Mild Mitral Regurgitation

Video 6 Transesophageal Echocardiography Demonstrating a Single-Plane View of the Mitral Valve, With a Mobile Echodensity Consistent With Vegetation on the Mitral Valve Leaflet, Confirming Infective Endocarditis Despite Prior Negative Transthoracic Imaging

Video 7 Transesophageal Echocardiography Demonstrating a Bi-Plane View of the Mitral Valve, With a Mobile Echodensity Consistent With Vegetation on the Mitral Valve Leaflet

Video 8 Transesophageal Echocardiography With Color Doppler Demonstrating Moderate Mitral Regurgitation With an Anteriorly Directed Mitral Jet

Video 9 Transesophageal Echocardiography Demonstrating the Long Axis of the Aortic Valve With a Small Echodensity Observed on the Right Coronary Cusp of the Aortic Valve Measuring 0.34 cm × 0.23 cm That Moves With the Valve, Suggestive of an Aortic Valve Vegetation

Video 10 Transesophageal Echocardiography Demonstrating the Short Axis of the Aortic Valve With a Small Echodensity Observed on the Right Coronary Cusp of the Aortic Valve Measuring 0.34 × 0.23 cm That Moves With the Valve, Suggestive of an Aortic Valve Vegetation
